# Understanding the structure and function of bacterial expansins: a prerequisite towards practical applications for the bioenergy and agricultural industries

**DOI:** 10.1111/1751-7915.12377

**Published:** 2016-07-01

**Authors:** Claudia Martinez‐Anaya

**Affiliations:** ^1^ Departamento de Ingeniería Celular y BiocatálisisInstituto de BiotecnologíaUniversidad Nacional Autónoma de MéxicoAv. Universidad 2001, ChamilpaCuernavaca62210MorelosMéxico

## Abstract

Since the publication of a landmark article on the structure of EXLX1 from *Bacillus subtilis* in 2011, our knowledge of bacterial expansins has steadily increased and our view and understanding of these enigmatic proteins has advanced with relation to their structure, phylogenetic relationships and substrate interaction, although the molecular basis for their mechanism of action remains to be determined. Lignocellulosic material represents a source of fermentable sugars for the production of biofuels, and cell‐wall degrading activities are essential to efficiently release such sugars from their polymeric structures. Because expansins from fungi and bacteria seem to be required to properly colonize or cause disease to plant tissues, and because they share some characteristics with their plant counterparts for loosening the cell wall they have been seen as a promising tool to overcome the recalcitrance of these materials. However, microbial expansins activity is at best, very low compared with plant expansins activity. This revision analyses recent work on bacterial expansins structure, function and biological role, emphasizing our need to focus on their mechanism of action as a means to design better strategies for their use, in both in the energy and agricultural industries.

## Introduction

Plant biomass, in the form of lignocellulose, is an abundant material that is readily obtained from culture crops and garden wastes, wood, or from used processed fibres such as cotton or linen. Because these materials exist in excess, they represent a potential cheap source of fermentable sugars for bioconversion to ethanol, but cannot be fully exploited because of their recalcitrant nature, which brings with it a concomitant increase in production costs (Zhang and Lynd, 2004; McCann and Carpita, 2008; Pauly and Keegstra, 2008; Vogel, 2008; Chundawat *et al*., 2011; Álvarez *et al*., 2016). Many laboratories focus their efforts in the manufacture of degradative enzymes of grater and/or longer lasting activity, however, the search for accessory proteins that through non‐enzymatic processes enhance the hydrolytic action of enzymes is another active area towards a more effective saccharification process (Allgaier *et al*., 2010; Smith *et al*., 2012; Ekwe *et al*., 2013; Singhania *et al*., 2013; Koeck *et al*., 2014; Lambertz *et al*., 2014).

Accessory proteins could potentially exploit various mechanisms: reducing the non‐productive adsorption of enzymes to the substrate; localizing activities into the more susceptible areas or hot‐spots of the cell wall; or the more desired quality of decreasing the crystallinity of cellulose and therefore increasing the modifiable surface area accessible to degrading enzymes (Arantes and Saddler, 2010). One such example is expansins that loosen the cell wall to allow tissue expansion and other processes for which cell wall modification is required (Cosgrove, 2016). Plant expansins belong to two main groups: α‐expansins that bind xyloglucan‐enriched cellulose (McQueen‐Manson and Cosgrove, 1995; Cosgrove, 2016), and β‐expansins that act on cell walls with high content of arabinoxylan (Sampedro *et al*., 2015). Bacterial expansins, on the other hand, can bind whole cell walls or cellulose (depending on their electrostatic nature, see below), but their productive substrate appears to be cellulose (Lee *et al*., 2010; Wang *et al*., 2013; Olarte‐Lozano *et al*., 2014); thus, laboratories around the world have analysed the effect upon cellulose recalcitrance following exposure to expansins. These efforts, although promising, have been hampered because, with the exception of a few of cases (explained below), plant expansins are difficult to produce in active form from heterologous systems leaving many questions unanswered about their mechanism of action. Bacterial homologues of the plant expansins have served as a model to study these proteins, nevertheless differences in the activity level between microbial and plant expansins reveal important differences, probably due to structural variations (Kerff *et al*., 2008; Georgelis *et al*., 2011, 2014). Small advantages in saccharification of bacterial expansin‐treated lignocellulose also indicate that although somewhat useful, their activity is far from useful, probably because of our inability to establish the best reaction conditions, especially as we still have not characterized how they perform their function or the role they play *in vivo* during plant**–**microorganism interaction. This review briefly analyses reports on the application of bacterial expansins in the treatment of lignocellulose, but underlines studies directed towards discerning the molecular basis of their activity, and the role they play during microbe‐plant interactions that will eventually allow proposing better uses for this proteins.

## Expansin use in saccharification

Expansins substrate locates in the plant cell wall, and different studies indicate a number of substrates depending on the expansin type, being one of them cellulose. However, expansins contrary to other cell wall modifying activities, disrupt the weak bonding between polysaccharides in the cell wall and weaken filter paper (composed exclusively of cellulose). This observation has prompted investigations into the use of *Bacillus subtilis* Exlx1 and expansins from other bacteria for lignocellulose exploitation under the premise that degradative enzymes would perform better on a modified, less recalcitrant cellulose, with a subsequent and much needed reduction of biofuel production costs. Indeed, after the identification of *B. subtilis* Exlx1 as an active expansin (Kerff *et al*., 2008), later research has reported higher conversion yields from different materials treated with combinations of cellulases and preparations of recombinant bacterial expansins, generally purified from *Escherichia coli*. However, the improvement is at best modest with an average increase of activity of approximately twofold (reviewed in Liu *et al*., 2015; Georgelis *et al*., 2015; Cosgrove, 2016); in the cases where the fold‐enhancement was greater such as that reported by Bunterngsook *et al*. (2014), the authors did not include an irrelevant protein as a control (BSA for instance) to correct for unspecific effects on cellulase activity. Cellulose enhancement also happen in drastic reaction conditions, such as high expansin concentration (up to 600 μg ml^−1^) with a very low cellulase loading (< 0.5 FPU); suggesting that expansin influence could result not from loosening of the substrates, but by cellulose enhancement through non‐specific mechanisms. Crystalline cellulose, filter paper and natural lignocellulosic materials have been used as substrates for reactions containing expansin and cellulase, and the results of experiments by a number of groups have been summarized before (Liu *et al*., 2015). The fact that synergy was observed only in the presence of very low cellulase activity reveals a flawed approach for the combined use of expansins and cellulases, given that the enhancement of hydrolytic activities is lost not only by increasing enzyme concentration but also by increasing expansin concentration, representing an important problem for industrial scaling. Georgelis *et al*. (2015) have proposed possible mechanisms of non‐specific cellulase enhancement such as increased cellulase stability, decrease of non‐productive binding to the substrate, or by expansins acting as surfactants given their hydrophobic and hydrophilic nature. In summary, the absence of synergism between expansins and cellulases to render significant conversion rates imply that our model of bacterial expansin action is incomplete.

The difficulty of expressing recombinant plant expansins has delayed comparative studies with bacterial expansins in the context of cellulase activity enhancement. Yet the favourable properties of plant expansins (greater activity and reproducible determinations) make continued efforts to establish heterologous expression systems for plant expansins worthwhile. One such attempt reported the expression of *Lycopersicum esculentum* ExpA2 in *Pichia pastoris*. And although (Liu *et al*., 2014) observed filter paper swelling after incubation with the recombinant expansin, enhancement of cellulase activity could only be detected at extremely low enzyme loading (0.004 U mg^−1^ filter paper) which was essentially abolished once cellulase loading reached 0.016 U mg^−1^ filter paper. Similar results were obtained when crystalline cellulose was treated with rice expansins ExpA4, ExpA33, ExpB8, ExpB12 and ExlA3 expressed in *E. coli* (Seki *et al*., 2014).

A promising technique to obtain substantial quantities of plant expansins is the use of heterologous plant expression systems, as shown by Yoon *et al*. (2015) that expressed the cucumber expansin gene *Cs*ExpA1 in maize seeds in combination with a high throughput functional screening assay based on cellulase activity enhancement. Targeting expression to the endoplasmic reticulum resulted in good yields, and assays with pretreated corn stover and pretreated tobacco stems gave similar results, either with native or recombinant *Cs*ExpA1, of an approximately 2.5‐fold increase of cellulase activity. In comparison with bacteria or yeast expression systems, this is a laborious technique that involves genetic manipulation of different generations of plants, however, the authors’ estimate a half reduction in costs of crude fungal cellulases by the use of this expression system when considering expansin concentration and stability (among other advantages) produced in maize seeds. Furthermore, even when biochemical analysis was not the authors’ objective, expansin production in such large amounts opens many possibilities for their characterization.

## Expansins activity and measurement

**Figure 1:**
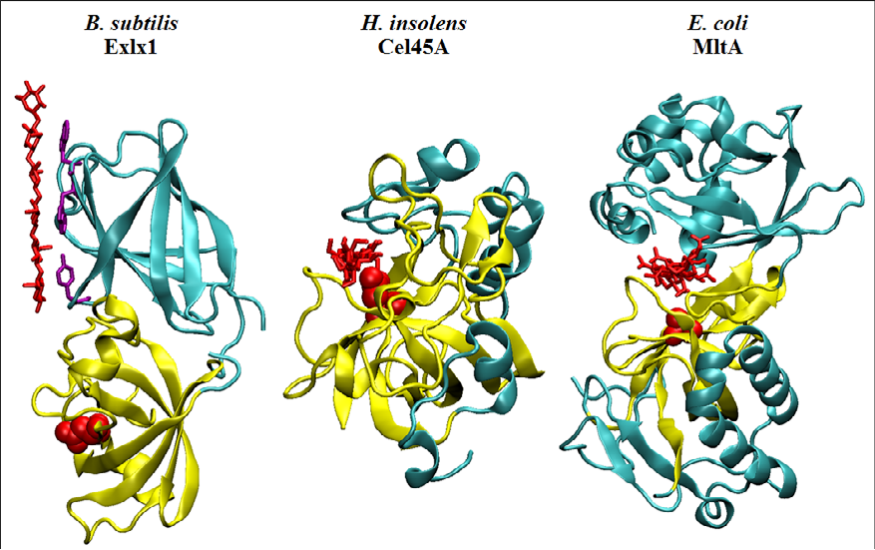
Crystal structures of proteins sharing a six‐stranded double psi beta barrel fold (yellow) interacting with oligosaccharide molecules (red). *Bacillus subtilis* Exlx1 (PDB: 4FER) shows a glycan chain interacting through its aromatic residues (purple) on the surface of D2. *Humicola insolens* Cel45A (PDB: 4ENG) forms an active site residing along the enzyme's surface where a cellohexaose molecule (viewed from the side) is stabilized by hydrogen bonds with residues forming the catalytic cleft. *Escherichia coli* MltA (PDB: 2PI8) shows an enzyme monomer complexed with a chitohexaose molecule (also viewed from the side) that interacts at the interface between its two domains, which is tightly bound in a deep and narrow active site groove. Active aspartates D82 and D121 (from *Bs*Exlx1 and *Hi*Cel45A, respectively), and the position of the active D308 of EcMltA (mutated by an alanine in this structure) are highlighted in red.

Through the eighties and nineties, Daniel Cosgrove determined the effects of plants expansin activity on the cell wall by devising an ingenious method to quantify two phenomena: (i) the elongation of plant tissue through induction of endogenous expansin expression at low pH or by adding exogenous expansins to a permeable, heat‐inactivated stalk fragment and (ii) the stress relaxation of a tensed cell wall sample upon expansin addition, both responses occurring rapidly (1–2 min) in comparison to glycosyl hydrolases (that take much longer to affect the cell wall) indicating a different mode of action (McQueen‐Manson and Cosgrove, 1995; Cosgrove, 1997). Domain 1 of expansins resembles hydrolytic enzymes because of a common fold (double psi beta barrel), which is the structure where the active site forms in different glycosyl hydrolases family 45 (Davies *et al*., 1996) or type 2 lytic transglycosylases (Van Straaten *et al*., 2005) in combination with other domains to shape a cleft of varying depth that accommodates and stabilizes the polysaccharide substrate (Fig. 1). Both protein families possess a conserved catalytic residue equivalent to Asp82 in expansins (this functional residue has a different position in other expansins, but for easy comparison the *B. subtilis* expansin numbering is indicated as a reference here, and throughout this review), which instead of being in a cleft is found at a shallow surface composed of conserved residues that interact with a glucan chain; however, many studies have shown that expansins are incapable of hydrolysis, and the current hypothesis states that expansin disrupts weak bonding between cell wall polymers allowing slippage of the stress bearing polymers (Mcqueen‐mason and Cosgrove, 1994; McQueen‐Manson and Cosgrove, 1995) (Cosgrove, 2005). The complexity of the cell wall structure has obstructed determining the mechanism of action of expansins, but another important limitation for their study is the lack of a standard method to quantify their activity, which is done by extensometry in a home‐made apparatus created by Daniel Cosgrove (Durachko and Cosgrove, 2009); however, an extensometer is difficult to construct and calibrate with precisely adjusted parameters for proper comparisons in other laboratories around the world. Thus, only a few bacterial expansins have been analysed for creep activity and stress relaxation of plant tissues showing activity up to 10 times lower when compared with expansins from plants, while for more reliable and robust determinations the samples require pretreatment with sodium hydroxide (Kerff *et al*., 2008; Georgelis *et al*., 2011, 2014). A partially successful alternative is offered by the use of a tensometer (Universal Testing Machine, Instron) that possesses the necessary sensitivity to measure the force required to break the material under test. This technique has been used to show that expansins from *Hahella chejuensis*,* Pectobacterium carotovorum* and *Clostridium clariflavum* weaken filter paper (Lee *et al*., 2010; Olarte‐Lozano *et al*., 2014; Artzi *et al*., 2016). It has been further shown that, for *P. carotovorum* expansin, activity depends on Asp82 and aromatic residues on D2 (Olarte‐Lozano *et al*., 2014). However, for measurements using a tensometer the materials must be removed from solution and mounted between the grips of the equipment, which is impractical for activity quantification under different conditions such as reaction buffer, temperature, etcetera; and more importantly, making real time determinations impossible to perform. Nevertheless, evidence obtained through tensometer measurements should be regarded as the minimum requirement for functional identification of bacterial expansin activity, at least until a calibrated extensometer becomes more widely available.

## Expansins targets

Plant and microbial expansins share a greater degree of structural similarity than their low sequence identity (approximately 15%) might suggest (Yennawar *et al*., 2006; Kerff *et al*., 2008). They are composed of two tightly juxtaposed domains that are stabilized by ionic interactions and in some cases by disulphide bonds (Kerff *et al*., 2008; Yennawar *et al*., 2013; Pastor *et al*., 2015). On the surface, a shallow groove comprised of conserved residues spans the two domains and forms the polysaccharide binding site, where a glucan molecule of nine saccharides contacts the substrate, stabilized by three aromatic residues on D2 and through polar interactions on D1. Expansins also contain a number of charged residues on the surface that, in the case of a positively charged expansin such as *Bs*Exlx1, serve to attach to acid moieties of pectin and hemicellulose (Kerff *et al*., 2008; Georgelis *et al*., 2011). On the other hand, acidic expansins such as Exl1 from *P. carotovorum* or Exlx2 from *H. chejuensis* seem to be repelled by pectin and hemicellulose and binding increases after their depletion (when cellulose is the main polysaccharide remaining), further supporting the notion that cellulose is the target of bacterial expansins (Lee *et al*., 2010; Olarte‐Lozano *et al*., 2014). This difference allows separation of bacterial expansins in two groups: acidic, when their pI < 5; or basic, when pI > 8 (Pastor *et al*., 2015). The reason for this is mysterious considering that productive binding occurs through the polysaccharide binding surface that is conserved among all groups of expansins. Indeed, binding parameters to Avicel are comparable for the basic *Bs*Exlx1 (*B*_*max*_ = 0.34 μmol g^−1^; *K*_*d*_ = 2.12 μM) and the acidic *Pc*Exl1 (*B*_*max*_ = 0.34 μmol g^−1^; *K*_*d*_ = 2.97 μM), despite having very different pIs (9.2 and 4.8, respectively), whereas binding of *Bs*Exlx1to whole cell walls is almost four‐fold higher than *Pc*Exl1 at pH 7.5. Because the regions where charge differences among expansins localize are outside the polysaccharide binding surface, it has been proposed that electrostatic binding to negative components of the cell wall for basic expansins (*Bs*Exlx1 being the prototype) is non‐productive, but could serve to sequester expansin activity to certain regions of the cell wall and probably to avoid hydrogen bond disruption in non‐desired locations (Kerff *et al*., 2008). In agreement with this, an *Bs*Exlx1 triple mutant (R173Q/K180Q/K183Q) with reduced positive charge decreases non‐specific binding to pectin and hemicellulose and shows a twofold increase in plant tissue elongation activity with respect to the wild type (Georgelis *et al*., 2011). Wang *et al*. (2013), then employed this hyper active mutant in sensitivity‐enhanced solid‐state NMR experiments, and observed a precise localization of expansin on the cell wall of *Arabidopsis thaliana*, in particular to cellulose domains with an altered crystal conformation probably due to a rich xyloglucan content and low pectin. In comparison, confocal microscopy experiments showed that Swollenin –a fungal non‐hydrolytic protein distantly related to expansins (Saloheimo *et al*., 2002)– locates to regions within dislocations of the cellulose fibre (Gourlay *et al*., 2015); these dislocations are structures found in the secondary cell wall of some plant species and contain higher levels of amorphous cellulose as revealed by co‐staining with a type B cellulose binding module (CBM) for which it presents affinity. These experiments suggest that Swollenin‐dependent deconstruction could begin at this sites (Gourlay *et al*., 2015). Equivalent experiments with bacterial expansins could help in determining whether these too exhibit preference for binding to particular structural conformations of cellulose fibres.

Although expansin binds to cellulose, an evident change to the structure of cellulose has not been reported to date despite efforts been made (Seki *et al*., 2014); this contrasts with *Kluyveromyces lactis* swollenin that affects the crystallinity index of substrates (as determined by X‐ray diffraction) varying from 10% reduction on filter paper up to 22% reduction on α‐cellulose (although no effect was observed with Sigmacell) (Jäger *et al*., 2011), suggesting that expansin effect occurs without modification of the crystal structure of cellulose, or that the modification is very subtle or very short‐lived.

## Expansin binding to cellulose

Expansins D2 binding to crystalline cellulose through its aromatic residues resembles the interaction of type A CBMs with cellulose (Kerff *et al*., 2008); but despite adopting a β‐sandwich fold (which is a common fold among CBMs), D2 has been recognized as an independent family of CBMs assigned to the CAZy database family 63 because of its low sequence similarity to CBMs from hydrolytic enzymes, its different biological role, and its absence of metal ions (Boraston *et al*., 2004; Georgelis *et al*., 2011). Indeed, *Bs*Exlx1 binds microcrystalline cellulose with an affinity of approximately 2 μM (either at 4°C or 25°C) that is comparable to the values obtained for CbpA (1.7 μM) or Ctcbd3 (2.5 μM), whereas type‐B or type‐C CBMs (which show affinity for amorphous cellulose and oligosaccharides, respectively (Boraston *et al*., 2004)) association constants are one order of magnitude weaker. Also, the maximum concentration of binding sites on Avicel are equivalent for *Bs*Exlx1 (0.11 μmol g^−1^ Avicel at 4°C) and type A CBMs such as CtCBD3 (0.34 μmol g^−1^ Avicel at 25°C). Binding to Avicel or bacterial crystalline cellulose is not affected by low temperature since this is a reversible entropy‐driven process (Georgelis *et al*., 2012). At protein concentrations between 20 and 25 μM, isotherms exhibit one binding site for expansin through D2, whereas binding to oligosaccharides is very weak (*K*_*d*_ > 1 mM). Crystallographic data of *Bs*Exlx1 bound to cellohexaose shows hydrophobic bonding of the aromatic residues W125 and W126 aligned to the plane of the pyranose rings of the carbohydrate, whereas Y157 interacts but overlaps inexactly with the glucan chain (Georgelis *et al*., 2012). It also shows that a hydrogen bond forms between K119 and residue G5 of the cellohexaose molecule, although a K119A mutant reduces binding to whole cell walls to a greater degree than binding to cellulose, indicating its importance for electrostatic binding (Georgelis *et al*., 2011, 2012). This weight of evidence points to the conclusion that the main function of D2 is to bind the substrate, whereas the loosening function depends on D1.

## Mechanism of action of expansins

Compared with the information available for the binding of expansins to a polysaccharide chain through D2, relatively little is known about the mode of action and interaction of the D1 domain upon the substrate. Mutagenesis experiments indicate a requirement for Asp82 for cell wall extension and filter paper weakening in *Bs*Exlx1 (Georgelis *et al*., 2011), and for filter paper weakening in Exl1 from *P. carotovorum* (Olarte‐Lozano *et al*., 2014). Other important D1 residues for creep activity are Thr14, Asp71 and TyrY73. Determination of the crystallographic structures of *Bs*Exlx1 in complex with cellohexaose failed to show D1 binding to this molecule, indicative of a weak interaction, in agreement with experiments where D1, although properly folded, was unable to bind Avicel or wheat coleoptile cell walls when expressed separately from D2 (Georgelis *et al*., 2011, 2012). Silveira and Skaf recently analysed the interaction of *Bs*Exlx1 with a glucan molecule through molecular dynamics simulations, with findings that corroborate the experimental data on D2 binding (with the exception of a planar interaction with the glucan chain instead of the twisted conformation around Tyr157 observed in the crystal structure) (Kerff *et al*., 2008; Silveira and Skaf, 2016). Interestingly, D1 interaction showed hydrogen bonding to a glucan molecule for Asp82 together with Thr14 (the two most persistent in the dynamics simulations), and Ser16 and Asp71. Asp82 had the strongest interaction (−30 kcal mol^−1^) inducing a twist in the glucan chain that was assisted by Thr12. Chain deformation by the firm hydrogen bond on Asp82 and further stabilization by the aromatic side chain of Tyr73 could serve as the basis for the activity of expansins, the authors suggest, since their simulations were able to explain the experimental data obtained with certain inactive *Bs*Exlx1 mutants. A mutation of Asp82 for Asn inactivates *Bs*Exlx1 creep activity (Georgelis *et al*., 2011), and the molecular dynamics indicates that this could be due to a more recurrent inactive conformation produced by the interference of the aromatic side chain of Tyr73 with Asn82, which prevents the Asn82 residue from forming a stable bond with the substrate. According with the authors, the D82N mutation would be reverted by a second mutation (Y73L) that in their simulations also induced a twist in the polysaccharide in a similar manner to the conformation attained in the wild type protein. An aromatic residue in position 73 is also important to provide conformational stability for the substrate‐contacting residues and to allow a conserved hydrogen bonding between Thr12 and Asp82 that is present in the structures of GH45 and type 2 lytic‐transglycosylases. This stability is affected in the Y73A mutant, which experimentally was also inactive (Georgelis *et al*., 2011). To investigate how universal the twisted saccharide chain model could be, it would be interesting to perform molecular dynamics simulations with the structure of *Clavibacter michiganensis* expansin (4JJO) that instead of a Tyr73 contains a Cys residue engaged in a disulphide bond with Cys69 (PDB: 4JJO, Yennawar *et al*., 2013).

**Figure 2:**
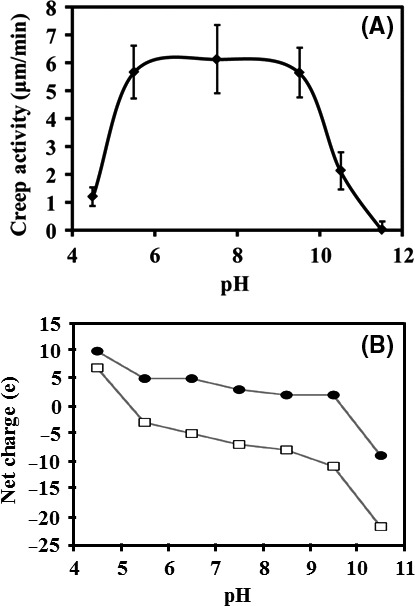
Experimental cell wall extension activity profile of *Bacillus subtilis* Exlx1 of alkali‐treated wheat coleoptiles (A), determined by Georgelis *et al*. (2011), is compared with the theoretical net charge of *Bs*Exlx1 (black circles), and of *Pectobacterium carotovorum* Exl1 (white squares) at different pH (B) (Olarte‐Lozano *et al*., 2014).

**Figure 3:**
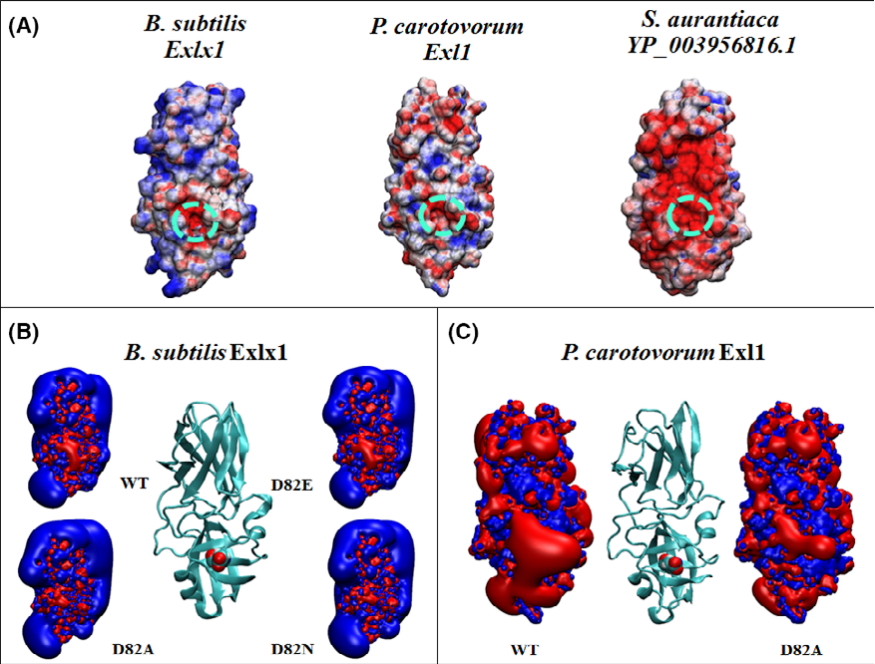
Electrostatic distribution of expansins. (A) Surface electrostatic potential of expansin structures from *Bacillus subtilis*,* Pectobacterium carotovorum* and *Stigmatella aurantiaca* (YP_003956816.1). The dotted circle indicates an electronegative area in the vicinity of the active Asp82 in *Bs*Exlx1 and *Pc*Exl1, which is replaced by a Leu at the equivalent position in *S. aurantiaca*. Colours by electrostatic potential mapped at the surface are red to blue from −5 kT/e to +5 kT/e. Isopotential contours of inactive mutants D82A and D82N expansins from *Bs*Exlx1 (B) and D82A from *Pc*Exl1 (C), showing a reduced influence compared with the electronegative Asp82 (Pastor *et al*., 2015).

As mentioned previously, bacterial expansins can be either acidic or basic due to protonable residues on their surface, and are therefore susceptible to pH changes. Analysis of creep for *Bs*Exlx1 showed a plateau of maximum activity between pH 5.5 and 9.5 (Georgelis *et al*., 2011) that nicely coincides with its predicted molecular net charge of around +5e at the same pH range, which changes abruptly to +10e below pH 5 or to less than −20e above pH 10 becoming too negative (Olarte‐Lozano *et al*., 2014); and although an equivalent experiment for an acidic expansin has not been performed, we could predict a similar behaviour where instead of a plateau we would find a broad peak with a maximum at approximately pH 7.5 (Fig. 2). Charge distribution and electrostatic profiling by domain, inside and outside the polysaccharide binding surface, were analysed by Pastor *et al*. (2015), revealing that D1 is electronegative, particularly in the vicinity of the functional Asp82, resulting in a polarization of the site with respect to the rest of the domain that occurs independently of the acidic or basic nature of the molecule (Fig. 3). Contour profiles of the inactive D82A mutants in either *Bs*Exlx1 (basic) or *Pc*Exl1 (acidic) show a reduced polarization that could explain their lack of function if this attribute was required for activity. In the case of the D82E mutant the electronegativity persists, but its activity is reduced to less than one‐half compared with the wild type expansin probably due to steric impediment due to an extra methyl group that could hamper the interaction with the glucan chain. This implies that an electronegative influence at the functional site would be necessary (rather than a negative charge *per se*), opening up the possibility for the existence of expansins with a functional residue different from Asp. Such is the case of the expansin from *Stigmatella aurantiaca* YP_003956816 (that possesses Leu instead of Asp but which still maintains an electronegative area similarly to other expansins; Pastor *et al*., 2015); although it should be noted that the experimental activity of this protein still awaits verification.

## Some bacterial expansins are modular proteins

Apart from the expansin module comprising D1 and D2, some microbial expansins are fused to other protein modules with homology to: family 5 glycosyl hydrolases (GH5 family), carbohydrate binding domains (cellulose binding domain or chitin binding domain), dockerin domains and discoidin (F5/8C) domains (Nikolaidis *et al*., 2014). Of these, experimental activity has been demonstrated for the N‐terminal GH5 catalytic domains of expansins from *Xathomonas campestris* and *C. michiganensis* that also contains an intermediate type 2 CBM; and also for their expansin modules that are active on pure cellulose (such as bacterial crystalline cellulose from *Gluconacetobacter xylinus* and filter paper) and facilitate extension of alkali‐pretreated wheat coleoptiles (Georgelis *et al*., 2014). Nevertheless, the biological function for these multi‐modular proteins is still unknown, given that the cellulase activity of *C. michiganensis* protein is unaffected by the removal of the expansin domain, supporting the notion that expansins and cellulases are non‐synergistic. Recently, Chen *et al*., 2015; analysed two expansins from the anaerobe *C. clariflavum* naturally encoded as fusion proteins with N‐terminal dockerin domains for possible interactions into the cellulosome (Artzi *et al*., 2014, 2015; Chen *et al*., 2015). However, for a reason not specified by the authors the native dockerin domain of both expansins (Clocl_1298 and Cocl_1862) was replaced with a dockerin domain from *Bacteroides cellulosolvens* allowing assemble into designer cellulosome, showing an increase of release of reducing sugars detected at low cellulase (cellulosome) concentration and short reaction time (Chen *et al*., 2015). Expansin expression of Clocl_1862 (named *Ccl*Exl1) but not of Clocl_1298 (*Ccl*EXL2) by *C. clariflavum* grown in microcrystalline cellulose‐containing medium was demonstrated by a more recent study of these proteins (Artzi *et al*., 2016), although *Ccl*Exl1 expression occurred at low levels (only 2% of the main scaffoldin, ScaA, found in the same cellulosome fraction). *Ccl*Exl1 interacts with type I cohesin‐containing scaffoldins of *C. clariflavum* and *Clostridium cellulolyticum* preferably, as opposed to cohesins from other species, thus confirming the functionality of the predicted dockerin domain. *Ccl*Exl1 shows affinity for crystalline cellulose over acid‐swollen cellulose, xylan, and showed little affinity towards wheat straw, as expected from an acidic expansin (pI 4.8). *Ccl*Exl1 weakened filter paper at higher levels than *Bs*Exlx1 or *Pc*Exl1, reducing the tensile force required to break a strip from 29% to 44% in comparison to untreated samples. As in previous reports, synergism was observed when a filter paper strip was incubated with the enzymes exoglucanase GH48‐Doc and endoglucanase GH9‐CBM3, also from *C. clariflavum*, but only after a previous incubation of one hour with an excess of *Ccl*Exl1 (17 mM of expansin to 0.5 μM of enzymes), since no synergy was observed when all proteins were incubated simultaneously, probably due to competition for binding sites within the substrate, as suggested by the authors. Also, the hydrolytic enhancement was lost when equimolar amounts of proteins (cellulases and expansin) were added to the reaction mixture for the hydrolysis of microcrystalline cellulose. Interestingly, the activity of different fractions of the *C. clariflavum* cellulosome (termed MCCI and MCCII) with 0.5 μM *Ccl*Exl1, increased irrespectively of the expansin addition timing (previous or simultaneous incubation), which suggests that other components of the cellulosome could be necessary for the expansin action on the substrate, although no increase of hydrolysis was observed after a twofold increase in concentration of the cellulosome fractions, pinpointing to a complex mechanism of action of *Ccl*Exl1 (Artzi *et al*., 2016).

Despite the functionality of extra domains related to cellulose utilization, enhancement of cellulose deconstruction by expansins has not been observed, therefore the reason for their existence as modular proteins deserves additional analyses.

## Biological role of expansins

Expansin‐containing bacteria are either Gram‐positive or Gram‐negative, and a correlation with the type of expansin they encode was found: acidic proteins are encoded mostly by Gram negative species, whereas the majority of basic expansins are found in the genome of Gram positive bacteria (Pastor *et al*., 2015). Whether this correlation has any biological implications needs further study. Unfortunately, very little literature exists that shed light upon the biological role expansins. Kerff *et al*. (2008), found that *Bs*Exlx1 binds avidly peptidoglycan, resisting treatment with 5 M NaCl before final release through heating, adding detergent and digesting with lysozyme; this strong interaction suggests that *Bs*Exlx1 could be attached to the bacterial cell, possibly aiding to its location to important sites within the plant cell wall. *Bs*Exlx1 is dispensable for peptidoglycan synthesis, as a deletion mutant strain contains normal quantities of muropeptides that are also indistinguishable from the wild type strain. Normal cell morphology, cell division timing and daughter cell separation are reported for the mutant, and only when the autolytic pathway is induced, the mutant strain showed reduced autolysis that is complemented by the expression of *Bs*Exlx1 from a plasmid, suggesting a participation in its breakdown. The major defect of the mutant strain was observed in maize root colonization experiments showing a dramatic reduction (> 80%) in comparison to the wild type cells, which shows the importance of the expansin activity for proper colonization (Kerff *et al*., 2008). Additional evidence for expansin involvement in microbe‐plant interactions comes from two early works that independently reported the study of natural plasmids, pCM1 from *C. michiganensis* subsp. *michiganensis* (Jahr *et al*., 2000) and pCS1 from *C. michiganensis* subsp. *sepedonicus* (Laine *et al*., 2000), each codifying for a modular endo‐β‐1,4‐glucanase CelA fused to a type II CMB and an expansin module at its C‐terminus (analysed by Georgelis *et al*. in the previous section). Transcription of CelA gene from pCS1 was observed, and deletion constructs lacking the expansin domain fail to cause disease symptoms in tomato plants despite a normal cellulase activity that is also required for disease (Jahr *et al*., 2000). It is tempting to suggest an evolutionary advantage for the presence of expansins in bacteria that infect via the plant vascular system given the remarkable fact that severe xylem‐invaders genre codify expansins *Clavibacter* (with two expansin genes), *Pectobacterium*,* Ralstonia*,* Xanthomonas* and *Xylella* (Nikolaidis *et al*., 2014; Pastor *et al*., 2015). In view of the weight of evidence counting against bacterial expansins function in cell wall modification, at least in a similar sense to plant expansins, an attractive possibility for their biological function would be localized in the xylem, which is composed of secondary cell wall where crystalline cellulose is abundant, functioning as molecular anchors for other activities –cellulase domains, for instance‐ which could be a possibility of the existence of at least this type of gene fusions.

Not all plant pathogens contain expansin genes, therefore it is possible that expansin‐containing bacteria may have developed an additional or alternative infection mechanism that is expansin‐dependent. Cosgrove's group propose that expansins were acquired by microbes from plants in ancient events of eukaryote to prokaryote horizontal gene transfer, and that this process could still be taking place because the expansin sequence of *Streptomyces acidiscabies* relates more to plant expansins than to bacterial expansins (Nikolaidis *et al*., 2013).

If expansins provide of evolutionary advantages to their species, why do all plant‐interacting bacteria not express expansin genes? Is it possible that cell wall loosening activities, similar in role but independent of the expansin family, await discovery? Proteomic experiments of cellulose rich environments would be one strategy for identifying potential candidates.

## Perspectives for bacterial expansin analyses

The current model of expansin activity suggests that once a productive interaction occurs between the expansin and its target, the expansin would move along the polymers rupturing hydrogen bonds under the pressure exerted by the expanding cells. This model works well for a healthy plant undergoing active growth, however, it is still too early to extrapolate this behaviour to the activity of bacterial expansins. Kinetics of expansin production by pathogens will tell us about the condition of the cell wall that expansins would encounter. If expansins are expressed soon after bacteria interacts with the plant, is the tensile stress from the plant cells also the driving force for microbial expansin function? On the other hand, if expansin expression is required in later stages of tissue maceration, in the case of an infection with highly degradative pathogens, might it be possible that less activity is required because expansin substrates are more exposed? It might be possible that differences on cell wall integrity are the reason for the low activity of bacterial expansins in comparison to their plant counterparts. Perhaps expansins, from beneficial bacteria, possess relatively little activity to avoid disorganizing the cell wall more than needed to preserve its structure to some extent or to prevent activating the plants defence mechanisms. Or could it be that low microbial expansin activity is a relic of their postulated plant origin, and their main function is still unknown, as suggested by others? Much work is still needed to gain information on the role of expansins during plant‐bacteria interactions, and to answer the above questions a number of methods must be used to determine their expression, localization and possibly interaction with other molecules apart from their known substrates. Analysis of expansins expressed by their organism of origin would help in answering the above questions, which eventually could facilitate the manipulation and redirection of the expansin use for our exploitation.
